# Impact of the X Chromosome and sex on regulatory variation

**DOI:** 10.1101/gr.197897.115

**Published:** 2016-06

**Authors:** Kimberly R. Kukurba, Princy Parsana, Brunilda Balliu, Kevin S. Smith, Zachary Zappala, David A. Knowles, Marie-Julie Favé, Joe R. Davis, Xin Li, Xiaowei Zhu, James B. Potash, Myrna M. Weissman, Jianxin Shi, Anshul Kundaje, Douglas F. Levinson, Philip Awadalla, Sara Mostafavi, Alexis Battle, Stephen B. Montgomery

**Affiliations:** 1 Department of Pathology, Stanford University School of Medicine, Stanford, California 94305, USA;; 2 Department of Genetics, Stanford University School of Medicine, Stanford, California 94305, USA;; 3 Department of Computer Science, Johns Hopkins University, Baltimore, Maryland 21218, USA;; 4 Sainte-Justine University Hospital Research Centre, Department of Pediatrics, University of Montreal, Montreal, Québec H3T 1J4, Canada;; 5 Department of Psychiatry, Stanford University School of Medicine, Stanford, California 94305, USA;; 6 Department of Psychiatry, University of Iowa Hospitals & Clinics, Iowa City, Iowa 52242, USA;; 7 Department of Psychiatry, Columbia University and New York State Psychiatric Institute, New York, New York 10032, USA;; 8 Division of Cancer Epidemiology and Genetics, National Cancer Institute, Bethesda, Maryland 20892, USA;; 9 Department of Computer Science, Stanford University, Stanford, California 94305, USA;; 10 Department of Statistics, University of British Columbia, Vancouver, British Columbia V6T 1Z4, Canada

## Abstract

The X Chromosome, with its unique mode of inheritance, contributes to differences between the sexes at a molecular level, including sex-specific gene expression and sex-specific impact of genetic variation. Improving our understanding of these differences offers to elucidate the molecular mechanisms underlying sex-specific traits and diseases. However, to date, most studies have either ignored the X Chromosome or had insufficient power to test for the sex-specific impact of genetic variation. By analyzing whole blood transcriptomes of 922 individuals, we have conducted the first large-scale, genome-wide analysis of the impact of both sex and genetic variation on patterns of gene expression, including comparison between the X Chromosome and autosomes. We identified a depletion of expression quantitative trait loci (eQTL) on the X Chromosome, especially among genes under high selective constraint. In contrast, we discovered an enrichment of sex-specific regulatory variants on the X Chromosome. To resolve the molecular mechanisms underlying such effects, we generated chromatin accessibility data through ATAC-sequencing to connect sex-specific chromatin accessibility to sex-specific patterns of expression and regulatory variation. As sex-specific regulatory variants discovered in our study can inform sex differences in heritable disease prevalence, we integrated our data with genome-wide association study data for multiple immune traits identifying several traits with significant sex biases in genetic susceptibilities. Together, our study provides genome-wide insight into how genetic variation, the X Chromosome, and sex shape human gene regulation and disease.

Many human phenotypes are sexually dimorphic. In addition to males and females having recognizable anatomic and morphological differences, accumulating evidence suggests that they exhibit differences in the prevalence, severity, and age of complex diseases. Classic examples of sex-biased diseases include autoimmune disorders (Whitacre et al. 1999; Whitacre 2001), cardiovascular disease (Lerner and Kannel 1986; Mendelsohn and Karas 2005), cancer susceptibility (Cohn et al. 1996; Naugler et al. 2007), and psychiatric disorders (Breslau et al. 1997; Pigott 1999; Hankin and Abramson 2001). While genetic factors may underlie observed differences, determining the genetic contribution to sexual dimorphism has generally lagged behind the hormonal contribution due to challenges in both study design and statistical power (Luan et al. 2001; Patsopoulos et al. 2007; Ober et al. 2008). Despite these limitations, several studies have discovered genotype-by-sex interaction effects in human phenotypes, such as anthropometric traits (Heid et al. 2010; Randall et al. 2013), bone mineral density (Liu et al. 2012a), complex diseases (Liu et al. 2012b; Myers et al. 2014), and intermediate cellular phenotypes such as gene expression (Dimas et al. 2012; Yao et al. 2014). To explain the etiology of these sexually dimorphic traits, several mechanisms have been proposed, including those arising due to the X Chromosome (Dobyns et al. 2004; Ober et al. 2008).

Although genome-wide association studies (GWAS) have uncovered numerous loci associated with complex phenotypes on the autosomes, the X Chromosome is significantly underrepresented in such work. Indeed, only one-third of GWAS include the X Chromosome, largely due to specialized analytical methods required for processing and interpreting genetic data on this chromosome (Wise et al. 2013). Furthermore, many large-scale functional genomic studies investigating the effect of genetic variants also exclude the X Chromosome (Dimas et al. 2009; Montgomery et al. 2010; Pickrell et al. 2010; Lappalainen et al. 2013; Battle et al. 2014; The GTEx Consortium 2015). Motivated by the underutilization of the X Chromosome, recent studies have characterized the role of the X Chromosome in the heritability of human phenotypes (Chang et al. 2014; Tukiainen et al. 2014). However, no studies to date have systematically investigated the contribution of the X Chromosome in the context of both regulatory variation and its interaction with sex.

By leveraging a recent, large genetic study of gene expression (Battle et al. 2014), we comprehensively survey the impact of sex and genetic variation on the X Chromosome on human gene expression to improve our understanding of the genetic and molecular basis of sex-biased disease risk. Our study overcomes several limitations of previous eQTL and sex-specific eQTL studies which have either ignored the X Chromosome, conducted analyses in cell lines which may inaccurately reflect in vivo sex differences (Dimas et al. 2012), had insufficient power to detect sex-specific eQTLs (Trabzuni et al. 2013), or focused on only specific variants for sex-specific eQTL analysis (Castagne et al. 2011; Yao et al. 2014). We extend these studies to describe the characteristics of eQTL on the X Chromosome versus the autosomes, address the relationship between sex-specific gene expression and chromatin accessibility, and identify the contribution of multiple eQTLs to informing sex-biased disease risks. Together, our study provides new insight into the genome-wide regulatory mechanisms of sexual dimorphism and the importance of including the X Chromosome and sex in the design, analysis, and interpretation of genetic studies.

## Results

To study sex-specific genetic variation in humans, we obtained gene expression data for the Depression Genes and Networks (DGN) cohort comprised of 922 individuals of European ancestry across the United States (Battle et al. 2014; Mostafavi et al. 2014). Gene and isoform expression were quantified from whole blood 51-bp single-end RNA sequencing data (Methods). Each individual was also genotyped for 737,187 single nucleotide polymorphisms (SNPs) located on the autosomes and X Chromosome on the Illumina HumanOmni1-Quad BeadChip and then imputed using the 1000 Genomes Phase 1 reference panel (Methods).

### Sex-specific expression variation across the genome

**Figure 1.**
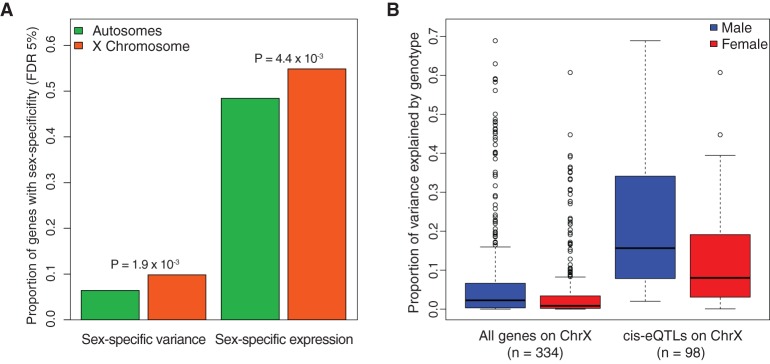
Differential expression variance within the sexes. (*A*) Comparison of genes with significant sex-specific expression variance (FDR 5%) and sex-specific expression (FDR 5%) on the autosomes and the X Chromosome in the DGN. To test for differences in mean expression and variance, the number of males and females were matched (*n* = 274). One-sided Fisher's exact test *P*-values *above* bars indicate significance of higher expression or variance on the X Chromosome relative to autosomes. (*B*) Proportion of variance explained (PVE) by genotype on the X Chromosome in males and females. To test for the PVE, the number of males and females were matched (*n* = 274). We tested all genes on the X Chromosome and genes with a *cis-*eQTL (Bonferroni adjusted *P*-value < 0.05).

Sex-specific patterns of gene expression have been well studied in the literature across many organisms (Yang et al. 2006; Ober et al. 2008; Reinius et al. 2008; Trabzuni et al. 2013). However, beyond differences in mean gene expression levels, differences in expression variation may be attributed to interaction effects of genomic variants and both environmental and biological variables, such as sex (Idaghdour and Awadalla 2012). We investigated both sex-specific patterns of gene expression levels and variance across the genome. After correcting for known technical covariates in the expression data (see Methods), we tested if gene expression variance in females is equivalent to that in males. We limited our analysis to genes expressed in both sexes and matched the number of males and females tested via random subsampling to ensure that sample size did not influence measured variance (Methods). We identified 924 genes with sex-specific expression variance (5% FDR). Across the genome, we observed that a higher proportion of genes with sex-specific variance are on the X Chromosome compared to autosomes (9.8% versus 6.4%; *P*-value = 1.9 × 10^−3^, Fisher's exact test) (Fig. 1A). This pattern remains the same for different normalization methods of the gene expression data and variance tests (*P*-value of Fisher's exact test equals 4.04 × 10^−3^ and 1.57 × 10^−4^ when the difference in variance is assessed using the F-test or Brown-Forsythe test, respectively) (Supplemental Figs. S1, S2), after correcting for gender effects on the mean (*P*-value = 1.57 × 10^−4^) and replicates in the ImmVar cohort providing cell-type–specific data (Ye et al. 2014) (*P*-value < 5.4 × 10^−3^) (Supplemental Fig. S3), indicating that this is unlikely to be an artifact of differences in cell-type proportions between sexes. As with variance, we observe more genes on the X Chromosome exhibiting sex-specific gene expression compared to autosomes (54.8% versus 48.4%; *P*-value = 4.4 × 10^−3^, Fisher's exact test) (Fig. 1A), an observation predicted by theory (Rice 1984) and seen in other species (Ranz et al. 2003; Yang et al. 2006). To help account for any potential bias in the relationship of variance and mean, we tested whether genes with sex-specific expression variance are genes with a significant difference in their mean expression between the sexes (FDR < 0.05, Welch's two-sample *t*-test) and detected no significant enrichment (*P*-value = 0.39, χ^2^ test).

To understand why the X Chromosome exhibits unique patterns of expression variance relative to the autosomes, we tested if the hemizygosity of the X Chromosome contributes to greater expression variance in males. To test this hypothesis, we estimated the proportion of expression variance explained by common genetic variants (MAF > 0.05) in males and females for each gene on the X Chromosome. We found that the mean estimated percentage of variance explained by genotype is 1.7-fold higher for males than females (2.3% versus 0.9%; *P*-value = 6.14 × 10^−5^, Wilcoxon rank-sum test) (Fig. 1B). This difference remained when restricting only to genes with a *cis*-eQTL (Bonferroni-adjusted *P*-value < 0.01; 21.0% versus 12.5%; *P*-value = 6.4 × 10^−5^, Wilcoxon rank-sum test) (Fig. 1B). In contrast, if we evaluate the proportion of variance explained by genotype on an autosomal chromosome, we observe no significant difference between males and females (*P*-value = 0.557 and 0.686, Wilcoxon rank-sum test) (Supplemental Fig. S4). We then asked if the autosomal genes with sex-specific expression variance are enriched in specific biological processes and found that these genes are enriched in cell death and regulation of apoptosis (Supplemental Table S1). These observations suggest that divergent regulation of cell death occurs between the sexes, supporting previous studies that have found sex-specific alterations in apoptosis within immune and neural cell populations (Molloy et al. 2003; Lang and McCullough 2008).

### Identification and characterization of X-Chromosome *cis-*eQTLs

**Figure 2.**
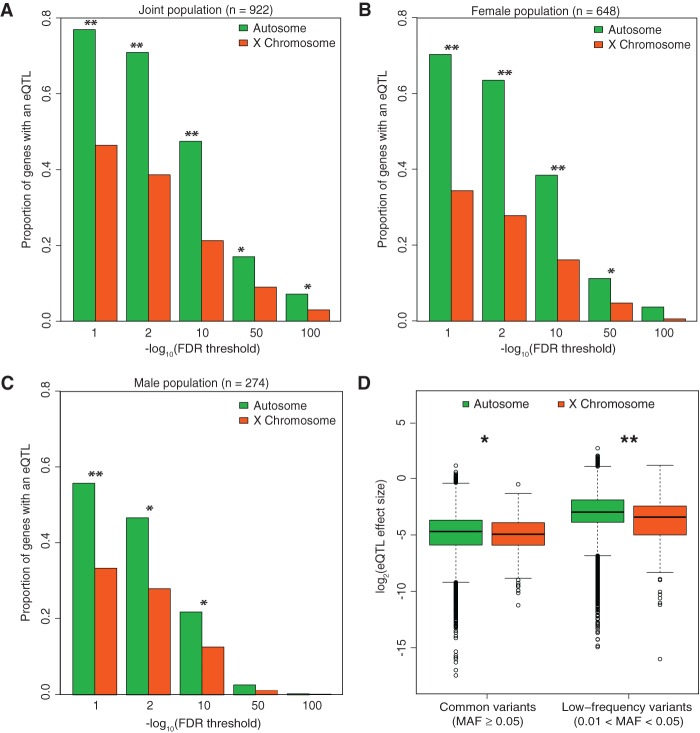
Characterization of eQTLs on the X Chromosome. (*A*) Proportion of genes with an eQTL at different FDR thresholds discovered in the joint (*n* = 922), (*B*) female (*n* = 648), and (*C*) male (*n* = 274) populations. (**) *P*-value < 1 × 10^−15^, (*) *P*-value < 0.05, Bonferroni adjusted χ^2^ test. (*D*) Comparison of eQTL effect size between autosomes and the X Chromosome for common variants (MAF ≥ 0.05) and low-frequency variants (0.01 < MAF < 0.05). The difference in effect size is statistically significant for both common ([*] *P*-value = 0.0382, two-sided Wilcoxon rank-sum test) and low-frequency variants ([**] *P*-value = 1.21 × 10^−7^).

We tested for *cis-*eQTL on the X Chromosome and autosomes using linear regression. At a genome-wide level FDR of 5%, we detect eQTLs on 74.8% of autosomal genes and 43.7% of X Chromosome genes. For a range of FDR thresholds, we find a depletion of eQTLs on the X Chromosome relative to autosomes (two-sided χ^2^ test, *P*-value = 9.4 × 10^−37^ at FDR 1%) (Fig. 2A). To determine if either male hemizygosity of the X Chromosome or female X-inactivation influenced this observed depletion, we also detected eQTLs in just the male and female populations alone. In both male and female populations, we observed a depletion of eQTLs on the X Chromosome across multiple FDR thresholds (*P*-value < 10^−15^, χ^2^ test) (Fig. 2B,C), compared to autosomal eQTL rates. The depletion of X Chromosome eQTLs further replicated in multiple individual cell types, including monocytes from the ImmVar cohort and lymphoblastoid cells from the Geuvadis cohort (Supplemental Table S2). To ensure that this depletion of eQTLs on the X Chromosome was not due to lower power in the X Chromosome compared to the autosomes, e.g., lower minor allele frequency (MAF) or mean gene expression, we compare the distributions of MAF and mean expression for all SNPs and genes that were considered in the eQTL analyses. We observe only a slightly smaller proportion of SNPs with 0.05 < MAF < 0.15 and genes with lower mean expression in the X Chromosome compared to the autosomes, suggesting that we are at least as powered to detect eQTLs in the X Chromosome as we are in the autosomes. We also compared the MAF, *F*_ST_, and expression levels of detected eQTLs and observed that X Chromosome eQTLs have lower MAFs (0.19 versus 0.23; *t*-test *P*-value = 3.8 × 10^−7^), higher *F*_ST_ (0.0847 versus 0.0287; *t*-test *P-*value = 0.0433), and slightly lower mean expression levels (*t*-test *P*-value = 2.0 × 10^−3^) relative to autosomal eQTLs (Supplemental Fig. S5A–C). Based on these observations, we can speculate that the X Chromosome's unique evolution and selective pressures, which are known to contribute to reduced genetic diversity compared to autosomes (Keinan et al. 2009; Gottipati et al. 2011) and affect dosage effects on expression levels (Gupta et al. 2006; Kharchenko et al. 2011), may contribute to the depletion of eQTLs.

#### eQTL effect sizes on the X Chromosome

To understand the properties of X Chromosome eQTLs, we compared effect sizes to those of autosomal eQTLs within females. For eQTLs detected from common variants (MAF ≥ 0.05), we observe that the effect size on the X Chromosome is 1.17-fold lower relative to autosomes (*P*-value = 3.8 × 10^−2^, Wilcoxon rank-sum test) (Fig. 2D). For eQTLs detected from low-frequency variants (0.01 < MAF < 0.05), we observe that effect size on the X Chromosome is 1.34-fold lower relative to autosomes (*P*-value = 1.2 × 10^−7^, Wilcoxon rank-sum test), suggesting increased purifying selection for large eQTL effects on the X Chromosome versus the autosomes.

Previous studies have found that hemizygosity of the X Chromosome in males may cause unusual patterns of evolution, including lower nucleotide diversity and more efficient removal of deleterious alleles on the X Chromosome compared to the autosomes (Andolfatto 2001; Schaffner 2004; Lu and Wu 2005; Vicoso and Charlesworth 2006). To test the relationship of eQTL effect size and purifying selection on the X Chromosome, we obtained the ratios of nonsynonymous to synonymous substitutions (*d*_N_/*d*_S_) of human-rhesus genes as an indicator of selective constraint, quantile binned the *d*_N_/*d*_S_ ratios, and compared the eQTL effect sizes on the X Chromosome to autosomes within each bin. For genes under the greatest degree of constraint (lower 20% quantile bin, *d*_N_/*d*_S_ 0–0.05), we observe significantly lower effect sizes on the X Chromosome (*P*-value = 3.6 × 10^−4^, Wilcoxon rank-sum test) (Supplemental Fig. S5D). For genes under less constraint (higher *d*_N_/*d*_S_ ratios), we observe similar but less significant patterns (*P*-value < 5.0 × 10^−2^). This evidence suggests that X Chromosome genes experience increased purifying selection on regulatory variation relative to the autosomes.

### Identification and characterization of sex-interacting *cis*-eQTL

**Figure 3.**
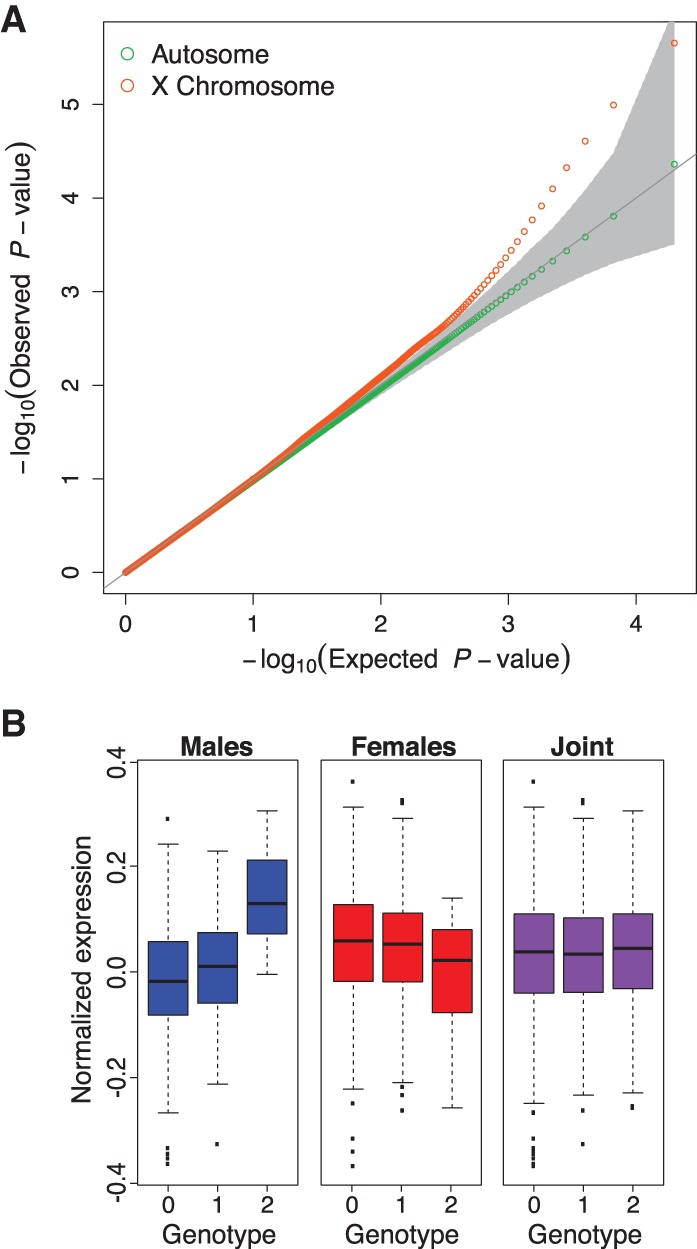
Discovery of sex-interacting eQTLs. (*A*) Quantile-quantile (QQ) plot describing the sex-interacting eQTL association *P*-values for SNPs tested within 1 Mb of genes on the X Chromosome (orange) and the autosomes (green) with 95% confidence interval (gray). (*B*) Sex-interacting eQTL (*q*-value = 0.0198) for *DNAH1* (dynein, axonemal, heavy chain 1), a protein-coding gene involved in microtubule motor activity, ATPase activity, and sperm motility.

To identify sex-interacting *cis*-eQTLs, we used a linear model with a genotype-sex interaction term. Genotype-by-sex interactions occur when the effect of genotype on expression differs between males and females. For example, the genetic effects on expression may be present in only one sex, may have different magnitudes of effect, or may even have opposing directions of effect in the two sexes (Supplemental Fig. S6). We observe an enrichment of sex-specific eQTLs on the X Chromosome compared to the autosomes (Wilcoxon rank-sum test, *P*-value = 8.2 × 10^−4^ and 1.9 × 10^−5^ for the top 50 and 500 associations, respectively) (Fig. 3A). After adjusting for the number of SNPs tested per gene using Bonferroni and subsequently identifying sex-interacting eQTLs using gene-level significance at 5% FDR, we discover six eQTLs (four autosomal and two X Chromosome eQTLs) with significant sex interactions (Table 1). If we restrict our tests to variants previously detected as *cis-*eQTLs (Bonferroni adjusted *P*-value < 0.1), we improve power but the total number of discoveries is unaffected (Supplemental Fig. S7). Notably, two of the six sex-interacting eQTLs discovered in our DGN cohort were also identified as sex-interacting eQTLs in other cohorts (Supplemental Table S3). Specifically, a genotype-sex interaction was previously identified for *NOD2* at rs9302752 (nominal *P*-value = 9.03 × 10^−13^) in the Framingham Heart Study (Yao et al. 2014); rs9302752 is in high linkage disequilibrium (LD) (*R*^2^ = 0.93) with the top sex-interacting eQTL variant we identified for *NOD2* in the DGN cohort (nominal *P*-value = 5.1 × 10^−10^). We also reanalyzed the genotype and expression data from the ImmVar (Ye et al. 2014) and CARTaGENE cohorts (Awadalla et al. 2013; Hussin et al. 2015) to test if our sex-interacting eQTLs replicated in other cohorts (Supplemental Table S3); we observed that the sex-interacting eQTL at Chr 11: 62735958:D-*BSCL2* replicated in the CARTaGENE cohort (*P*-value = 2.89 × 10^−3^), and the remaining variants were not testable due to expression levels or lack of replication.

**Table 1.**
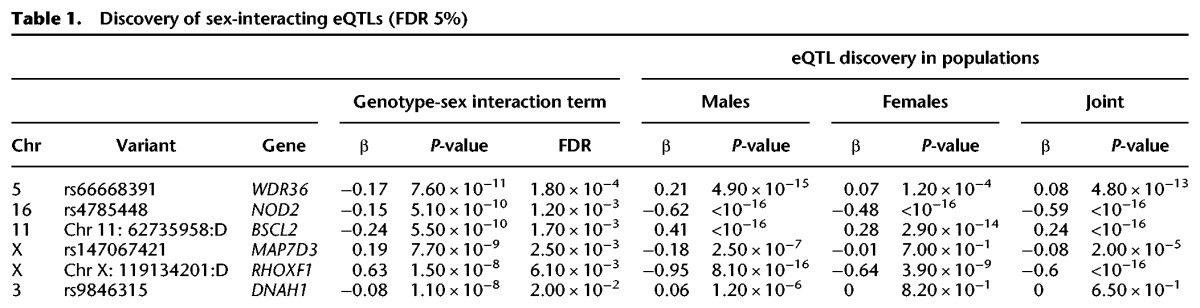
Discovery of sex-interacting eQTLs (FDR 5%)

We additionally used a binomial generalized linear mixed model (Knowles et al. 2015) to test for a sex-interacting allele-specific expression QTL (aseQTL). Specifically, we tested if alleles at heterozygous loci show allele-specific expression (ASE) and if the magnitude of ASE effects differs in males and females. Two of our six significant sex-interacting eQTL genes had heterozygous loci with sufficient read depth to measure allelic imbalance. Of these two genes, we observed that *NOD2* has a significant sex-interacting aseQTL (*P*-value = 2.5 × 10^−3^) (Supplemental Fig. S8A) and *DNAH1* has a possible sex-interacting aseQTL with small effect but falls slightly below the level of significance (*P*-value = 1.0 × 10^−1^) (Supplemental Fig. S8B), likely due in part to higher variance of allelic ratio observed at this locus. All variants tested for sex interactions in the DGN cohort can be browsed at http://montgomerylab.stanford.edu/resources.html.

Of the six genes with sex-interacting eQTLs, *DNAH1* on Chromosome 3 was distinct because this SNP-gene pair was not a significant eQTL in the female or joint population analysis (Fig. 3B). Although this gene exhibits similar expression between the sexes in whole blood (nominal negative binomial *P*-value = 0.81), it shows the highest expression in the testis compared to the remaining 46 tissues tested in the GTEx Project. We investigated this gene further and found that it is a force-producing protein with ATPase activity involved in sperm motility and flagellar assembly (UniProt ID Q9P2D7). The sex-specific function of this gene prompted us to test if the sex-interacting eQTLs were enriched in sex-specific biological processes. To evaluate this hypothesis, we tested the top 100 sex-interacting eQTLs for enrichment in genes involved in sex differentiation (GO:0007548) and discovered a modest enrichment (odds ratio = 6.6 [1.17–18.2], *P*-value = 4.3 × 10^−3^, Fisher's exact test). When testing all biological processes, no specific process passed multiple testing corrections; however, the top GO terms were linked to sex-specific biological processes (e.g., reproductive structure development and response to estrogen stimulus) (Supplemental Table S4).

To determine if sex-interacting eQTL discovery is driven by differences in gene expression between males and females, we tested for enrichment sex-interacting eQTLs within differentially expressed genes (FDR 10%). We observed no significant enrichment of genes with sex-specific expression for the top 100 sex-interacting eQTLs (odds ratio = 0.4 [0.05–1.58], *P*-value = 3.3 × 10^−1^, Fisher's exact test) compared to the background of genes expressed in whole blood. Concordant with a previous study (Dimas et al. 2012), this demonstrates that sex-interacting eQTLs likely do not arise as a consequence of expression differences between the sexes and may result from other factors that differ in a sex-specific matter, such as transcription factor activity, hormone receptors, and chromatin accessibility.

### Sex-specific chromatin accessibility

To identify the molecular mechanisms of sex-specific gene regulation, we generated and investigated differences in chromatin accessibility between males and females. We measured chromatin accessibility of peripheral blood mononuclear cells (PBMCs) from 20 individuals matched for age, ethnicity, and sex using the assay for transposase-accessible chromatin followed by sequencing (ATAC-seq) (Buenrostro et al. 2013). Using a negative binomial model, we identified 577 (0.69%) sex-specific chromatin accessibility regions at FDR 10% (see Methods).

**Figure 4.**
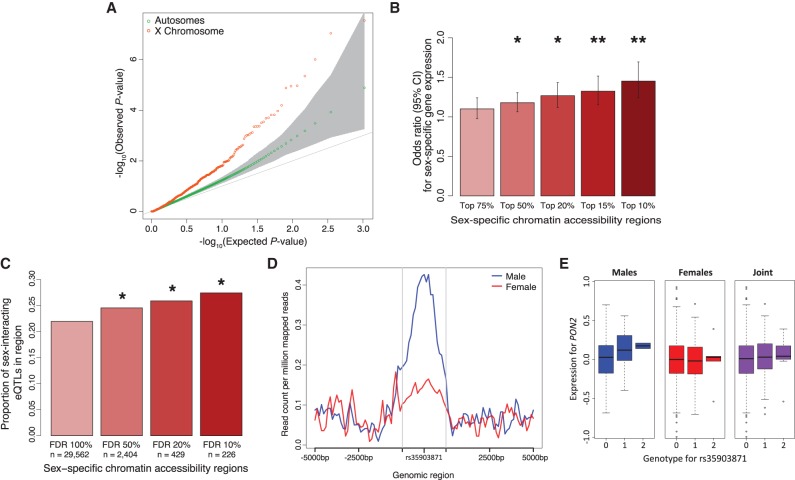
Discovery of sex-specific chromatin accessibility regions. (*A*) QQ plot for tests of differential chromatin accessibility between the sexes. 95% genome-wide confidence interval in gray. (*B*) Enrichment of genes with differential expression between the sexes (FDR 5%) with differential chromatin accessibility (varying thresholds) 40 kb upstream. (**) *P*-value < 10^−5^, (*) *P*-value < 10^−3^, Fisher's exact test. (*C*) Proportion of sex-interacting eQTL genes (*P-*value < 0.05) in differential chromatin accessibility regions (varying thresholds). (*) *P*-value < 5.0 × 10^−2^, Fisher's exact test. (*D*) Chromatin accessibility peak located at Chr 7: 95,063,722–95,064,222 and 5000 bp upstream and downstream. This region has differential chromatin accessibility between males and females (nominal *P-*value = 4.1 × 10^−4^, *Q*-value = 7.5 × 10^−2^). (*E*) Sex-interacting eQTL for *PON2* and rs35903871 located at Chr 7: 95,063,972 (nominal *P*-value = 8.0 × 10^−3^).

Considering the unique heterochromatic state of the X Chromosome in females, we asked if the distribution of sex-specific open chromatin regions differed between the autosomes and the X Chromosome (Fig. 4A). We observed an enrichment of sex-specific open chromatin regions on the X Chromosome (Wilcoxon rank-sum test, *P*-value = 3.5 × 10^−10^ and 1.4 × 10^−4^ for the top 50 and 500 associations, respectively). Following this observation, we hypothesized that regions on the X Chromosome are more likely to have greater chromatin accessibility in females due to genes escaping X-inactivation. Indeed, we found that X Chromosome regions are more likely to have higher chromatin accessibility in females than males compared to autosomal regions (odds ratio = 2.44 [1.99–3.01], *P*-value <2.2 × 10^−16^, Fisher's exact test) (Supplemental Fig. S9). To further interpret this, we then tested if the top sex-specific peaks on the X Chromosome are more likely to be genes escaping X-inactivation (Carrel and Willard 2005; Park et al. 2010). We observed that the top 10% of sex-specific peaks on the X Chromosome are enriched for genes escaping X-inactivation compared to genes in sex-shared peaks (44.6% versus 6.5%; odds ratio = 9.60 [2.70–34.49], *P*-value = 1.6 × 10^−4^, Fisher's exact test), indicating that a large number of the X Chromosome sex-specific peaks inform regions of escape from X-inactivation.

#### Integration of sex-specific chromatin accessibility with expression

One intuitive mechanism for sex-specific gene expression and sex-interacting eQTLs is sex-specific chromatin accessibility. Therefore, we sought to identify if differential open chromatin between the sexes was associated with sex-specific expression and genotype-sex interactions. First, we tested if genes with sex-specific expression (FDR 5%) are enriched in regions with sex-specific chromatin accessibility. We observed that genes with sex-specific chromatin accessibility 40 kb upstream of the gene TSS were associated with sex-specific expression (odds ratio = 1.45 [1.24–1.69], *P*-value = 4.7 × 10^−6^, Fisher's exact test) (Fig. 4B). We then investigated the directionality of enrichment and observed opposing directions of effect (i.e., genes with elevated expression in a particular sex were more likely to have depressed chromatin signal in the same sex and vice versa) (odds ratio = 2.38 [1.13–∞], Fisher's exact test *P*-value = 2.2 × 10^−2^). We speculate that genes with elevated expression may exhibit a depressed chromatin signal due to transcription factor occupancy or other chromatin modifications, as observed by others (Gilbert et al. 2004; Schneider and Grosschedl 2007; Natarajan et al. 2012).

Next, we investigated if sex-specific chromatin accessibility regions are enriched in sex-interacting eQTL genes by testing for genotype-sex interactions for variants within 1 Mb of the gene. We observe that genes with a sex-interacting eQTL (nominal *P*-value < 0.1) are likely to have differential chromatin accessibility between the sexes (odds ratio = 1.36 [0.99–1.81], *P*-value = 5.1 × 10^−2^, Fisher's exact test) (Fig. 4C). To ensure that we did not observe this enrichment by chance, we permuted the sexes for eQTL testing and tested sex-interacting eQTLs in sex-specific chromatin regions and observed no enrichment (Supplemental Fig. S10). We also investigated the directionality of enrichment and observed opposing directions of effect (i.e., eQTLs with elevated effect sizes in a particular sex were more slightly likely to have depressed chromatin signal in the same sex and vice versa) (odds ratio = 1.75 [1.19–∞], Fisher's exact test *P*-value = 5.8 × 10^−2^). As with sex-specific expression, eQTLs with higher effect in a particular sex may have decreased chromatin signal due to the occupancy of transcription factors at these regions (Gilbert et al. 2004; Schneider and Grosschedl 2007; Natarajan et al. 2012). In addition, we asked if sex-specific chromatin accessibility regions were enriched in sex-interacting eQTL variants. We tested the genotype-sex interaction term for the variant closest to the region mid-point and the closest five genes and observed weak but not statistically significant enrichment (Supplemental Fig. S11). One example of a sex-interacting eQTL in a sex-specific chromatin accessibility region is illustrated in Figure 4D, in which a SNP (rs35903871) is located in a region with greater chromatin accessibility in males (*P*-value = 4.1 × 10^−4^, FDR 7.5%) and exhibits genotype-sex interaction effects on *PON2* expression (β = 0.13; *P*-value = 8.2 × 10^−3^). If tested separately in each sex, this gene-SNP pair is an eQTL in males (*P*-value = 5.5 × 10^−3^) but not females (*P*-value = 5.0 × 10^−1^). Interestingly, *PON2* does not exhibit sex-specific gene expression (*P*-value = 6.3 × 10^−1^, FDR 89.2%) but has been associated with sex-specific effects in oxidative stress responses (Cheng and Klaassen 2012; Giordano et al. 2013; Polonikov et al. 2014).

### Integration of sex-specific effects with human disease

**Figure 5.**
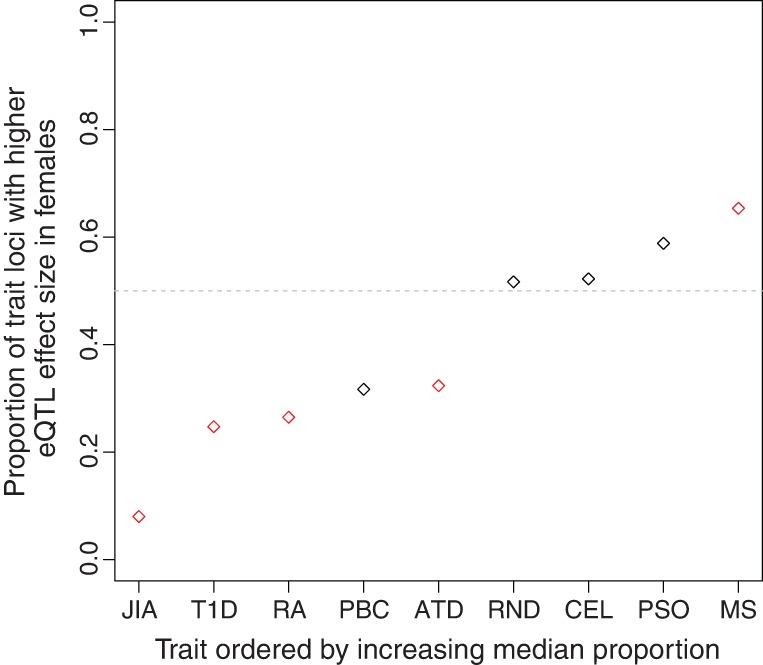
Disease associated variants with sex-biased eQTLs. Proportion of independent (LD-pruned) variants with higher eQTL effect sizes in females for GWAS variants of traits in ImmunoBase. Each trait variant tested had to be a significant eQTL variant (nominal *P*-value < 0.001). Red data points indicate traits with eQTL effect sizes that are significantly different between sexes (Bonferroni adjusted *P*-value < 0.05). (ATD) Autoimmune thyroid disease, (CEL) celiac disease, (JIA) juvenile idiopathic arthritis, (MS) multiple sclerosis, (PBC) primary biliary cirrhosis, (PSO) psoriasis, (RA) rheumatoid arthritis, (RND) random eQTL variants, (T1D) type 1 diabetes.

In humans, sexual dimorphism is observed in the incidence rates and severity of many common diseases, including cardiovascular, immune, and neurological diseases. For example, a recent study (Myers et al. 2014) identified six sex-specific asthma risk loci (*P*-value < 1 × 10^−6^), three of which exhibit genotype-sex interactions on expression in our study (nominal *P*-value < 0.05). Unfortunately, previous limitations on statistical power and study design have provided challenges for identifying significant genotype-sex effects in many disease association studies (Brookes et al. 2004; Wang et al. 2008). Given the role of regulatory variation in determining disease risk (Nica et al. 2010; Nicolae et al. 2010), we asked if genetic variants identified through genome-wide association studies have cumulatively different eQTL effects in males and females. To test this, we obtained trait-associated variants from ImmunoBase and examined the effect size of each GWAS variant on expression in males and females separately. We found that for the majority of autoimmune diseases, the disease-associated loci exhibit a bias in effect size in one sex (Fig. 5; see Methods). For example, a significant proportion of genetic variants associated with multiple sclerosis, a disease with well-documented sex-specific disparity in prevalence and severity (Whitacre et al. 1999; Whitacre 2001), exhibit a bias toward females (*P*-value = 5.6 × 10^−7^, binomial exact test). We performed an identical analysis using data available from the NHGRI-EBI GWAS catalog (Welter et al. 2014) and observe similar patterns (Supplemental Fig. S12). These observations indicate that disease-associated variants have different cumulative effects on gene expression in males and females underlying potential sex-biases in disease prevalence and severity.

## Discussion

In this study, we comprehensively evaluated the effect of the X Chromosome and sex on regulatory variation. First, we demonstrated that genes on the X Chromosome are more likely to have sex-specific expression compared to genes on the autosomes reflecting a study in mice (Yang et al. 2006) and a recent report in the human brain (Trabzuni et al. 2013). We observed similar differences for sex-specific gene expression variance between the X and autosomes, highlighting further interactions with sex. As expected, we identified that a portion of this difference was due to the hemizygosity of the X Chromosome in males where the exposure of individual alleles is not balanced by random X-inactivation. However, as with gene expression, sex-specific gene expression variance was exhibited genome-wide and we observed genes with higher sex-specific expression variance were more likely to be involved in apoptosis and regulation of cell death, consistent with previous reports highlighting sex differences in regulation of cell death (Du et al. 2004; Li et al. 2005; Jog and Caricchio 2013).

When considering the impact of genetic variation on gene expression for the X Chromosome versus autosomes, we observed a depletion of eQTLs and large eQTL effect sizes on the X Chromosome, supporting previous observations of more efficient purifying selection on the X Chromosome relative to the autosomes (Vicoso and Charlesworth 2006; Singh and Petrov 2007; Gottipati et al. 2011). In contrast, we tested the effect of genotype-by-sex interactions across the transcriptome and observed an enrichment of sex-interacting eQTLs on the X Chromosome. This increase reflects the increased proportion of sex-specific expression effects on the X Chromosome but also suggests the possibility that sex-specific eQTLs on the X Chromosome may not experience the same selective pressure as other eQTLs on the X Chromosome. To hone in on the mechanisms underlying sex-interacting expression and eQTLs, we generated chromatin accessibility data using ATAC-seq in 20 additional individuals (10 male and 10 female). Differences in chromatin accessibility are well known to influence gene expression and the exposure of genetic variants (Thurman et al. 2012). We identified that sex-specific chromatin accessibility was enriched for genes with sex-specific expression and for variants with genotype-by-sex interactions. However, we have so far not been able to identify any particular mechanism driving differential chromatin accessibility, despite preliminary evaluation of possibilities such as hormone receptor TF binding. This highlights a continued challenge and opportunity in mapping sex-dependent regulatory effects.

We discover that complex traits may have variants with cumulatively different effects between sexes, leading to potential sex-biases in their disease prevalence and severity. Previous analyses within the Framingham Heart Study specifically focused on identifying which GWAS variants exhibit genotype-by-sex interactions and discovered 14 of 11,672 tested had significant effects (Yao et al. 2014). This suggests that strong sex-specific genetic effects on gene expression may not underlie many complex trait-associated variants. Here, by considering the cumulative effect sizes of eQTL between sexes, we were able to identify well-known sex-biased diseases, suggesting that accumulation of sex-biased genetic disease risk could be polygenic across a broad range of variants and genes.

Many challenges and opportunities remain in the study of the combined effects of genetics and sex on gene expression. Indeed, despite correction of hidden covariates, blood is a heterogeneous tissue where differences in cell type and diverse environmental factors may still confound discovery of differences that can be causally attributed to sex. These challenges must be weighed against in vitro studies of sex differences that may not reflect hormone biology or other in vivo factors that expose meaningful differences in the activity of regulatory variants between sexes. As studies of diverse tissues increase their sample sizes (The GTEx Consortium 2015), we expect future studies will have comparable power to broadly expose the characteristics of X Chromosome and sex-specific eQTLs across a range of tissues. Moreover, in our study, the detection and interpretation of differences in expression variances between the two genders is complicated by the fact that the means of the two gender distributions are not equal (Sun et al. 2013). We attempt to overcome this issue by regressing out the mean effect of gender and testing for variance effects in the gender-corrected residuals. However, despite this correction, residual gender effects might still be present, if, e.g., gender has a nonlinear effect on the mean, and thus the biological and/or clinical significance of such differences in variances will always need to be determined on nonstatistical grounds. Further, our study identifies differences in purifying selection for eQTL on the X Chromosome versus the autosomes; however, as many common variants are likely neutral, future studies will benefit from considering low frequency and rare regulatory variants, as it is increasingly evident that personal genome interpretation will require careful integration of X-Chromosome biology between sexes.

## Methods

### Study cohort for expression analysis

In the discovery of associations between genotype, expression, and sex, we used the Depression Genes and Networks cohort (Battle et al. 2014; Mostafavi et al. 2014). The cohort is comprised of 922 individuals (648 females and 274 males) of European ancestry between the ages of 21 and 60 yr within the United States. A detailed description of the recruitment and phenotype data for this cohort is provided elsewhere (Battle et al. 2014; Mostafavi et al. 2014). See Supplemental Methods for details on processing of genetic and transcriptome data.

### Differential gene expression and variance analysis

Differential expression analysis was conducted using a negative binomial model in the R package DESeq (Anders and Huber 2010) to identify genes with sex-specific expression (FDR 5%) in a matched number of males and females (*n* = 274). Using DESeq, the samples were corrected for a number of technical factors previously identified (Battle et al. 2014; see Supplemental Methods).

To detect genes with sex-specific expression variance, we used technical factor-corrected and sample-matched data (*n* = 274). This was further compared to regression with PEER residuals. We applied three different testing strategies. First, we tested for differences in variance between males and females using an *F*-test. Second, we tested for normality of gene expression traits using the Shapiro-Wilk normality test. If normality was not rejected, we tested for differences in expression variance between males and females using an *F*-test; otherwise, we tested for differences in expression variance using the Brown-Forsythe Levene-type test. In the last approach, we quantile-normalized the log-transformed PEER residuals to a N(0,1) and ran a linear regression with quantile-normalized log-transformed PEER residuals as response and gender as a covariate. Again, if the normality assumption for the regression residuals was not rejected, we tested for differences between males and females in regression residual expression variance using either an *F*-test or a Brown-Forsythe Levene-type test. We used here the regression residuals, instead of the original response, to distinguish between genes that show genuine DV (since mean effects are removed in residuals) and genes that show DV due to DE.

### *cis*-QTL mapping

We performed association testing for expression QTL using the R package Matrix eQTL (Shabalin 2012), after correction for hidden covariates (Supplemental Methods; Supplemental Fig. S13). Variants within 1 Mb upstream of the transcription start site (TSS) and 1 Mb downstream from the transcription end site (TES) of a gene were tested using a linear regression model and accounting for sex as a covariate. We model the association between a candidate variant and gene expression by a linear regression:

$$y=\mu +\beta_{1}\times \mathrm{genotype}+\beta_{2}\times \mathrm{sex}+\varepsilon ,$$

where *y* denotes the observed expression level of the gene, μ the mean expression level across the population, β_1_ the regression coefficient of genotype, β_2_ the regression coefficient of sex, and ɛ ∼ N(0, $\;\sigma_{\varepsilon }^{2}$). In the primary QTL analysis, we only tested variants with MAF ≥ 0.05. In the low-frequency QTL analysis, we tested variants with MAF in the range 0.01–0.05. To control for multiple testing, we used Bonferroni correction to account for the number of variants tested per gene, retained the best association per gene, and controlled for FDR at the gene-level significance by the Benjamini-Hochberg method (Benjamini and Hochberg 1995).

### Identification of sex-interacting QTLs

We tested for genotype-by-sex (G×S) interactions in the full sample (*n* = 922) using the interaction model in the R package Matrix eQTL (Shabalin 2012). This model was used to test for equality of effect sizes between males and females. We model the interaction between genotype and sex by adding an interaction term to the linear regression:

$$\begin{array}{rl} y & =\mu +\beta_{1}\times \text{genotype}+\beta_{2}\times \text{sex}+\beta_{3}\\ & \;\times (\text{genotype}\times \text{sex})+\varepsilon , \end{array}$$

where *y*, μ, β_1_, and ɛ are as above, and β_2_ and β_3_ the regression coefficient of sex and the interaction between genotype and sex. For each gene, variants within 1 Mb upstream of the transcription start site (TSS) and 1 Mb downstream from the transcription end site (TES) of a gene were tested. We required variants to have a MAF ≥ 0.05 in the male and female samples. To remove cases where genotype is collinear with sex, we excluded variants with a rank less than three between genotype and sex. All sex-interacting eQTLs tested in the DGN cohort can be visualized at http://montgomerylab.stanford.edu/resources.html.

### Sample collection for open chromatin profiling

We obtained buffy coat samples from 20 healthy donors from the Stanford Blood Center (Stanford, CA). Male and female donors were equally represented for downstream differential analyses. To control for age and ethnicity, the samples selected were restricted to Caucasians between 18 and 45 yr of age. Age, ethnicity, and healthy status were self-reported by donors. ATAC-seq was applied to isolated PBMCs, and differentially accessible peaks were quantified (see Supplemental Methods).

### Differential open chromatin accessibility analysis

To identify regions with differential chromatin accessibility between males and females, we used the R package DESeq (Anders and Huber 2010). The data were normalized by the effective library size, and variance was estimated for each group. We tested for differential chromatin accessibility using a model based on the negative binomial distribution. To account for multiple testing, we adjusted the *P*-values by the Benjamini-Hochberg method; regions with a FDR < 0.05 were considered to be regions with differential open chromatin. We also evaluated the effect of removing hidden factors using SVA on the differential chromatin accessibility analysis and observed no differences in the number of significant peaks identified at multiple thresholds (*P*-value = 0.2, 0.1, 0.01, 0.001). To ensure that %GC bias and sequencing depth did not influence the differential chromatin accessibility analysis, we confirmed that there were no significant differences in these metrics between the sexes (Supplemental Fig. S14).

### Trait analysis

We obtained trait-associated SNPs from ImmunoBase (available at www.immunobase.org; accessed on April 2, 2015) and the NHGRI-EBI GWAS catalog (Welter et al. 2014) (available at www.ebi.ac.uk/gwas; accessed on April 2, 2015). We only considered independent variants (*R*^2^ < 0.5) with a genome-wide association threshold of *P*-value <1 × 10^−8^. Independent variants were selected using an LD pruning strategy such that the most strongly associated GWAS SNP for each trait for each LD block (*R*^2^ < 0.5) was selected. In the DGN cohort, we calculated the absolute effect size of each independent GWAS variant on expression for genes within 1 Mb in the male (*n* = 274) and randomly subsampled female (*n* = 274) samples. We considered a GWAS variant to have a sex-specific effect if the effect size in one sex was 1.2-fold greater than the opposite sex. For variants with a 1.2-fold greater effect in either sex, we ran a two-sided binomial exact test to determine if one sex had greater effects than the opposite sex. Only traits with at least 20 significant GWAS variants were considered in the trait analysis (Fig. 5; Supplemental Fig. S15).

## Data access

Genotype, raw RNA-seq, quantified expression, and covariate data for the DGN cohort are available by application through the National Institute of Mental Health (NIMH) Center for Collaborative Genomic Studies on Mental Disorders. Instructions for requesting access to data can be found at the NIMH Repository and Genomics Resource (RGR; https://www.nimhgenetics.org/access_data_biomaterial.php), and inquiries should reference the “Depression Genes and Networks study (D. Levinson, PI).” Open chromatin accessibility data from this study have been submitted to the NCBI Gene Expression Omnibus (GEO; http://www.ncbi.nlm.nih.gov/geo/) under accession number GSE69749.
